# Associated predictor covariates of cervical cancer stage and impact on survival at Khartoum oncology hospital, Sudan

**DOI:** 10.12688/f1000research.43590.2

**Published:** 2022-10-07

**Authors:** Amanda Elgoraish, Ahmed Alnory

**Affiliations:** 1 Epidemiology, Tropical Medicine Research Institute, National Centre for Research, Khartoum, Khartoum, P.O. Box 1304, Sudan; 2 Applied Statistics and Demography, Faculty of Economics and Rural Development, University of Gezira, Medani, Gezira, P.O. Box 20, Sudan

**Keywords:** Cervical cancer, survival, stage, Cox model, Sudan

## Abstract

**Background:** Cervical cancer can be invasive and advanced at diagnosis causing devastating suffering and premature death. The cancer stage at presentation is related to survival evaluation and several factors determine stage. The aim of the study was to examine predictors covariates associated with cervical cancer stage at diagnosis and its impact on patient prognosis and survival.

**Methods:** This retrospective cross-sectional study was carried out at Khartoum oncology hospital, Sudan. Participants were 239 cervical cancer patients diagnosed and treated between 2011-2015. Patients’ pathological and socio-demographic data were extracted from their medical files and survival times were calculated from follow-up. Chi-square, Kaplan-Meier, Log-rank test and Cox regression model were used to examine relationships between demographic and clinical variables and survival outcome.

**Results:** The mean age of the participants was 56.91 years and the majority were ≥45 years. Cancer survival analysis showed that the stage at diagnosis had limited association with socio-demographic factors, except where patients reside. Multivariate regression using the Cox proportional hazard model confirmed strongly that stage (p=0.035), chemotherapy (p=0.000) and radiotherapy (p=0.001) were the most likely predictor covariates of patient prognosis and survival time.

**Conclusions:** The results of this study suggest cancer stage at diagnosis and certain treatments are the most important factors impacting the prognosis and survival of patients with cervical cancer. Early detection and vaccination of women against HPV infection provide enormous opportunities for early diagnosis, more effective treatment and better chances of survival.

## Introduction

Cancer is a global public health problem, particularly in low- and middle-income countries, due to aging populations as well as broader social and environmental factors such as infectious diseases, education and ethnicity. ^1^ There are observed disparities in global cancer prognosis as mortality is higher among developing countries due to a lack of comprehensive early detection and effective medical care. ^2^ Cancer is a leading cause of death among women in both developing and developed countries and is increasing. ^3^ Women in developing countries develop the disease during their prime reproductive period and face more suffering from the disease complications and risk of death. ^4^ The most common cancers afflicting women are those of the breast and cervix. These cancers are closely related to sexual and reproductive behaviour in Woman. ^4^

Cervical cancer is a considerable cause of death among women in developing countries though it is preventable. It is, also, potentially curable if detected early and treated effectively. It is the second most commonly diagnosed cancer in women in developing countries. ^4^ In these countries, cancer mortality exceeds that of diseases related to death in pregnancy. However, there is clear diversity of trends among world regions, within regions and individual countries, in the incidence and mortality of cervical cancer. In Africa, there is a wide variation due to different exposure and disease susceptibility. ^1^ In Sub-Saharan Africa, the incidence is low but mortality rates are high due to advanced stage at presentation. ^5^ In Sudan, cervical cancer represents more than 16% of all cancer in women and 85% of cases are diagnosed at an advanced stage. ^6^^,^^7^ Cervical cancer is closely related to human papillomavirus (HPV) 16/18 infection and 78% of cases in the Sudan are diagnosed as invasive Lesions. ^8^ Moreover, the incidence and mortality rates of this invasive cervical cancer have increased during the last decade, especially among relatively young women. ^3^ This increase can be attributed to major changes in demography, economic and social factors, other disease risk factors and disease awareness. ^9^

Cancer burden and disparity among countries and people can be explained by prevalence, incidence and mortality, but the most direct measure of disease severity can only be provided by survival rates. ^10^ Early detection and prevention are the most effective ways to reduce premature death from cervical cancer; however, from a short-term perspective, immediate and effective treatment is the optimal solution. ^11^

Analysing cancer survival rates is an important way of discovering potential measures to be taken to improve the chances of better prognosis and survival. Cancer survival varies widely among different countries of the world due to differences in early detection and treatment modalities. By examining cancer survival from preventative measures and early detection, one can assess factors that have the greatest impact on cancer patient survival. Several studies have attempted to explain the relationships between patient survival and stage at diagnosis. These studies came to different conclusions about the strength and shape of these relationships and their impact. Researchers have a found significant association between the stage of cancer at diagnosis and survival. Socio-demographic attributes such as age, education, gender and ethnicity have also been shown to have some effects. ^12^ On other hand, differences in the type of treatment and quality of medical services might have an important effect on survival outcome. ^11^

Previous literature has shown the complexity of determining the drivers of international differences in the incidence and mortality of cervical cancer. It is most likely that each step in a patient’s journey to seek treatment contributes to some extent to these variations. ^12^ Many factors have been suggested to explain these variations; however, there is no complete agreement on potential predictor covariates that give overall explanations. Nevertheless, stage at diagnosis, tumor features and effective treatment have been postulated as the most widely accepted predictor covariates explaining degree and extent of their impact on prognosis and survival. For variations in cancer severity and survival, the stage at diagnosis remains the strongest predictor of cancer survival. ^13^ One can conclude that stage at diagnosis is related to survival evaluation and assessment. Several factors determine stage at diagnosis, including age, education, occupation, location, tumor features, availability and accessibility of adequate diagnostic and treatment facilities. ^11^ The stage at diagnosis is crucial to disease treatment as treatment plans are usually based on the stage of the disease. ^14^ The aim of this study was to examine predictor covariates associated with cervical cancer stage at diagnosis and its impact on cancer patient prognosis and survival.

## Methods

### Study design, setting and population

This was a retrospective cross-sectional hospital-based study. It was carried out at Khartoum oncology hospital, Sudan, which is the only medical institution providing complete diagnostic and cancer treatment services, where more than 80% of all Sudan cancer patients are registered. ^15^ Available patient information was collected from the hospital’s medical records during the study period from 2011-2015.

The target population of the study was patients with cervical cancer at Khartoum oncology hospital. To be included in the study, patients had to be between 18-79 years, be registered at the hospital, have complete medical records, have histopathologically confirmed cervical cancer and had received available treatment. Patients with incomplete medical records, unclear diagnosis and not treated at the hospital were excluded from the analysis. Written consent was obtained from the hospital to use participants’ data. No direct contact was made with patients during this data collection level. However, consent was obtained from participants during the active follow-up period.

The total number of patients at the hospital during the study period who met inclusion criteria, and were included in the analysis, was 239. This sample size of randomly selected participants was calculated from the number of cervical cancer patients among all cancer patients at this hospital as follows:

*The formula n = 3.84 p(1-p)/(precision)2*

*Proportion = 0.044 (report of Federal Ministry of Health 2015), precision=0.026 with 95% CI*

n = 3.84*0.044(1-0.044)/(0.026) ^2^ = 239

### Data collection and sources

The study data collected from Khartoum oncology hospital patients’ medical files were checked and rechecked for accuracy, duplication, completeness and consistency by the researcher with continuous assistance from the hospital medical staff. Active follow-up was carried out during the year 2016 by the researcher by contacting patients or next of kin to ensure collection of needed information concerning patients survival status data(dead or alive). Moreover, a checklist was prepared by the researcher from the literature on cancer patients’ survival concerning socio-demographic and clinical factors affecting survival to assist in needed data collection. ^16^^,^^33^ Data collected were tabulated and coded according to the American Joint Committee on Cancer (AJCC) and the Union for International Cancer Control (UICC) TNM staging system for analysis. ^17^

### Variables

Data collected concerning socio-demographic characteristics and clinical status of patients included age at diagnosis, level of education, occupation, marital status, urban/rural residential area, tribe, menopausal status, cancer stage at diagnosis, tumor grade, tumor cell differentiation, histological subtype, treatment modalities, residence state and close family relation with previous disease experience. Dates of birth, death, loss to follow-up, diagnosis and survival times were checked by using other information provided by hospital medical and statistical staff. This information was clearly defined in medical terms concerning certificate of death, confirmation of diagnosis and calculation of survival time.

### Statistical analysis

The statistical analysis is divided into two parts, descriptive statistics and regression analysis, using Stata version 11 (StataCorp, College Station, Texas) software. In the descriptive analysis, the visual presentation of data in tables and figures given, provides socio-demographic and clinical data in numbers, percentages, chi ^2^ and p-values and figures given provide clear indication of study population data distribution, relationships and associations. Then, important statistical conclusions were drawn. Statistical methods such as chi ^2^, Kaplan-Meier, log-rank test and Cox regression were used to find out most prognostic factors associated with cancer patient survival. Socio-demographic variables and stage at diagnosis were tested by chi ^2^. Stage, treatment, age and menopausal status were tested by log-rank test for equality. Socio-demographic variables, stage and treatment were tested by Cox regression. Stage was tested by Kaplan-Meier for survival rate between early and advanced levels. The analysis focuses on the stage at diagnosis as the most crucial prognostic predictor of cervical cancer patient survival.

## Results

### Descriptive statistics

The total number of patients included in the analysis was 239 ( Table 1). ^32^ The median age of participants was 56.91 years (SE=0.88, 95%CI=55.17-85.65). The majority of participants 82.9% were ≥45 years old. In total, 94.6% of participants were married, 92.5% were unemployed, and 97.9% were illiterate and/or had no formal education. Most participants resided in the western, Khartoum and eastern states of Sudan.

**Table 1.** Correlation between stage at diagnosis and socioeconomic variables among cervical cancer patients.

Variables	Total N(%)	Stage N(%)		Chi ^2^, P-value
		Early	Advanced	
Age group				
<30	4(1.7)	1(1.2)	3(1.9)	5.33, 0.255
30-44	37(15.5)	19(22.6)	18(11.6)	
45-59	81(33.9)	27(32.1)	54(34.8)	
60-74	91(38.1)	28(33.3)	63(40.6)	
≥75	26(10.9)	9(10.7)	17(11.0)	
Urban/Rural status				
Rural	60(25.1)	20(23.8)	40(25.8)	0.12, 0.734
Urban	179(74.9)	64(76.2)	115(74.2)	
Resident states				
Khartoum	49(20.5)	11(13.1)	38(24.5)	11.23, 0.047 *
Central	28(11.7)	16(19.0)	12(7.7)	
Northern	11(4.6)	2(2.4)	9(5.8)	
Eastern	32(13.4)	13(15.5)	19(12.3)	
Western	93(38.9)	33(39.3)	60(38.7)	
Southern	26(10.9)	9(10.7)	17(11.0)	
Tribes				
Non Arab descent African	150(62.8)	54(64.3)	96(61.9)	0.41, 0.814
Arab descent African	62(25.9)	22(26.2)	40(25.8)	
Other tribes	27(11.3)	8(9.5)	19(12.3)	
Education				
Illiterate	195(81.6)	72(85.7)	123(79.4)	1.59, 0.450
Low education	39(16.3)	11(13.1)	28(18.1)	
High education	5(2.1)	1(1.2)	4(2.6)	
Marital status				
Un married	13(5.4)	4(4.8)	9(5.8)	0.12, 0.734
Married	226(94.6)	80(95.2)	146(94.2)	
Occupation				
Non-employed	221(92.5)	77(91.7)	144(92.9)	0.12, 0.729
Employed	18(7.5)	7(8.3)	11(7.1)	
Menopause status				
Premenopausal	66(27.6)	28(33.3)	38(24.5)	2.12, 0.149
Postmenopausal	173(72.4)	56(66.7)	117(75.5)	
Parent relationship				
First degree relation	200(83.7)	75(89.3)	125(80.6)	3.37, 0.186
Relatives	22(9.2)	6(7.1)	16(10.3)	
Non relatives	17(7.1)	3(3.6)	14(9.0)	
Total	239	84(35.1)	155(64.9)	

* P-value<0.05 statistically significant association.

The distribution frequency of cervical cancer cases, according to the tumor, node and metastasis (TNM) staging classification system demonstrated that the majority 64.9% of participants were of advanced stage (III & IV), invasive squamous cell carcinoma 98.0%, with a high probability of spreading to distant organs ( Table 2). Most of these patients’ tumors were of high grade and moderately to poorly differentiated cells. Furthermore, most of these patients had first-degree relations with previous disease history. Regarding treatment, 76.6% of these patients received radiotherapy, 57.3% chemotherapy, 10.5% hormone therapy and 6.3% surgery, alone or in combination with other therapies.

**Table 2.** Test of equality of survival distribution for predictor variables.

variable	no. of subjects (%)	Mean of survival time (months)	95% Confidence interval (CI)	log rank (chi ^2^)	P-value
Stage ^a^					
I	9(3.8)	16.06	12.46 to 19.65	33.49	0.000**
II	75(31.4)	39.60	31.06 to 48.14		
III	110(46.0)	28.27	22.88 to 33.67		
IV	45(18.8)	13.09	6.55 to 19.62		
Early	84(35.1)	40.49	32.28 to 48.71	7.91	0.005*
Advanced	155(64.9)	23.77	19.32 to 28.23		
Treatment					
Surgery	15(6.3)	34.64	20.31 to 48.98	0.47	0.491
Chemotherapy	137(57.3)	40.13	33.45 to 46.80	19.12	0.000**
Radiotherapy	183(76.6)	33.86	28.4 to 39.31	3.63	0.057
Hormonal	25(10.5)	30.06	16.54 to 43.58	0.01	0.909
Age group					
<30	4(1.7)	29	28.9 to 29.01	10.13	0.038*
30-44	37(15.5)	27.22	17.31 to 37.17		
45-59	81(33.9)	33.85	27.72 to 39.98		
60-74	91(38.1)	29.26	21.83 to 36.68		
≥75	26(10.9)	11.92	7.01 to 16.82		
Menopause status					
Premenopausal	66(27.6)	30.16	22.44 to 37.89	0.26	0.611
Postmenopausal	173(72.4)	31.06	25.42 to 36.71		
Total	239	32.02	26.92 to 37.12		

^a^ % of invasive squamous cells carcinoma (98.0%), moderately to poorly differentiated cell (75.0%).

There was no significant correlation between age group of participants and stage (p-value>0.05), though the most frequent group among advanced stage was ≥45 years group. There was no significant correlation between cancer stage at diagnosis and other socio-demographic variables except state of residence (chi ^2^=11.23, df=5, p=0.047). This could be explained by the fact that Khartoum and nearby states have diagnostic and treatment facilities.

### Regression analysis

**Figure 1.**
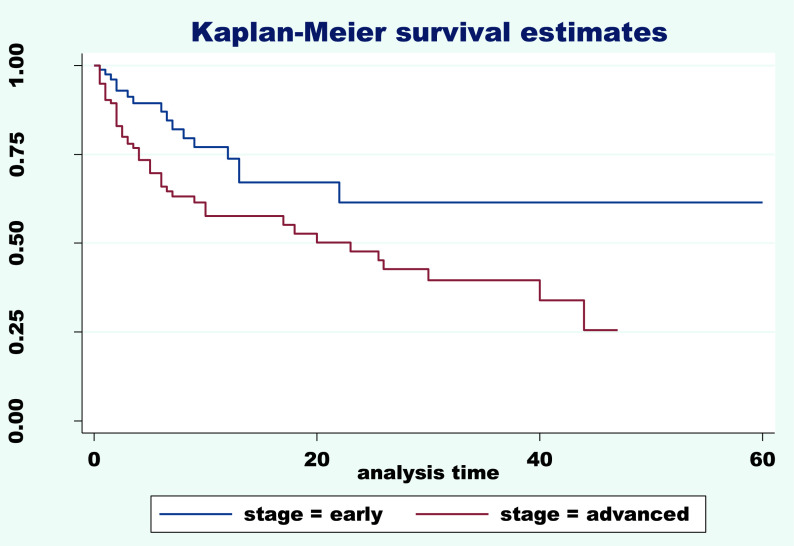
Survival rate according to early and advanced stage.

The overall mean survival time after 60 months of follow-up from time of diagnosis to the end of the study period was 32.0 months (95% CI=26.92 to 37.12). The lowest mean survival time according to stage levels was recorded at 13.1 months for stage IV(95% CI=6.55 to 19.62) ( Table 2). Moreover, the log-rank test when performed to compare and explain survival distribution clearly showed highly statistically significant differences between various levels of the stage at diagnosis (chi ^2^=33.49, df=3, p=0.000). Furthermore, the survival curve ( Figure 1) gives a visual description of these differences in survival times of different stage levels. The Kaplan-Meier and log-rank tests were performed according to early and advanced stages and indicated clear differences in survival time means between the two groups. A low mean survival time of 23.77 months at the advanced stage was observed compared to 40.49 months at the early stage. The chi ^2^ was 7.91, df=1 with p=0.005 ( Table 2). The graph of the two survival function curves was statistically different for the two groups. The lowest probability of 30% was recorded at the advanced stage ( Figure 1).

The Kaplan-Meier method and log-rank test were performed on the main four treatment therapies and only chemotherapy showed a highly statistically significant impact of chemotherapy on survival time. The chi ^2^ was 19.12, df=1 with p=0.000. As for age groups and survival times, the analysis revealed there was a clear difference in the age group ≥ 75 years. The log-rank test equals 10.13, df=4 and p=0.038. However, when the comparison was made according to their menopausal status, the results showed the difference was not statistically significant ( Table 2).

### Cox proportional hazard model

The Cox proportional hazards model was performed in four phases. ^18^ In the first univariate model, single predictor covariates; stage, treatment modality (chemotherapy) and age were statistically significantly associated with survival time ( Table 3) while other factors were not. The hazard ratio of advanced stage at diagnosis was more than twice that at an earlier stage (HR=2.18 at 95% CI=1.24 to 3.83, p=0.007). This large difference was highly statistically significant with P-value <0.05. In the second multivariate (adjusted) model, all predictor covariates were included simultaneously which showed that advanced stage at diagnosis, treatment (chemotherapy and radiotherapy), state (eastern and western) and urban status were the only predictor covariates of survival time. Then, in the third model, all non-significant predictors, except age and surgery, were dropped from the model. The third model showed that stage and treatment (chemotherapy and radiotherapy) were statistically significant predictor covariates. The hazard ratio which measures the risk of dying from cervical cancer was nearly two times at the advanced stage compared to the early one (HR=1.84, at 95% CI=1.05 to 3.26, p=0.035). Other covariates were not statistically significant. However, the final multivariate model confirmed strongly stage, chemotherapy and radiotherapy, after age and surgery were dropped from the model, were the most likely predictor covariates of survival times and cervical cancer patient prognosis and survival outcome ( Table 4).

**Table 3.** Univariate and multivariate regression models for association between survival time and predictor variables.

Factor	Univariate model		Multivariate model	
	HR(95%CI)	P-value	HR(95%CI)	P-value
Age	1.02(1.002 to 1.04)	0.027 *	1.02(0.99 to 1.05)	0.131
Stage				
Early	1(reference)	-	1(reference)	-
Advanced	2.18(1.24 to 3.83)	0.007 *	1.84(1.003 to 3.39)	0.049 *
Treatment				
Surgery	0.75(0.32 to 1.74)	0.497	0.52(0.19 to 1.38)	0.187
Chemotherapy	0.35(0.21 to 0.57)	0.000 **	0.23(0.13 to 0.43)	0.000 **
Radiotherapy	0.57(0.32 to 1.03)	0.063	0.29(0.14 to 0.62)	0.001 **
Hormonal	1.04(0.52 to 2.11)	0.910	1.14(0.48 to 2.71)	0.768
Residence state				
Khartoum	1(reference)	-	1(reference)	-
Central	0.51(0.19 to 1.30)	0.158	0.44(0.16 to 1.21)	0.111
Northern	1.44(0.59 to 3.53)	0.420	0.56(0.21 to 1.49)	0.243
Eastern	0.57(0.21 to 1.55)	0.267	0.23(0.06 to 0.82)	0.024 *
Western	0.67(0.35 to 1.27)	0.221	0.46(0.22 to 0.99)	0.049 *
Southern	0.97(0.44 to 2.14)	0.935	0.81(0.34 to 1.98)	0.649
Urban/Rural status				
Rural	1(reference)	-	1(reference)	-
Urban	0.78(0.46 to 1.32)	0.356	0.35(0.18 to 0.69)	0.002 *
Education				
Illiterate	1(reference)	-	1(reference)	-
Low education	1.52(0.81 to 2.86)	0.189	1.67(0.82 to 3.40)	0.158
High education	1.55(0.38 to 6.39)	0.543	2.42(0.55 to 10.70)	0.246
Marital status				
Un married	1(reference)	-	1(reference)	-
Married	1.69(0.52 to 5.45)	0.383	1.38(0.42 to 4.60)	0.593
Tribe				
Non Arab descent African	1(reference)	-	1(reference)	-
Arab descent African	1.42(0.85 to 2.39)	0.183	1.67(0.76 to 2.71)	0.158
Others	0.91(0.39 to 2.16)	0.834	1.56(0.62 to 3.95)	0.343
Occupation				
Non employed	1(reference)	-	1(reference)	-
Employed	0.98(0.39 to 2.44)	0.961	1.40(0.49 to 3.97)	0.526
Menopause status				
Premenopausal	1(reference)	-	1(reference)	-
Postmenopausal	1.16(0.65 to 2.09)	0.615	0.65(0.25 to 1.73)	0.391

HR: Hazard ratio, CI: confident interval.

* P-value<0.05 statistically significant association.

** P-value<0.001 highly statistically significant association.

**Table 4.** The final multivariate Cox model for association between survival time and predictor variables.

Factor ^a^	HR(95%CI)	P-value
Stage		
Early	1(reference)	
Advanced	1.84(1.05 to 3.26)	0.035 *
Chemotherapy	0.28(0.16 to 0.49)	0.000 **
Radiotherapy	0.33(0.17 to 0.64)	0.001 **

prob>chi ^2^=0.000, log likelihood=-293.99.

* P-value<0.05 statistically significant association.

** P-value<0.001 highly statistically significant association.

^a^ age, surgery and hormonal were dropped from the model.

Finally, one of the main assumptions of the non-parametric Cox proportional hazard model is proportionality upon which the Cox model and log-rank test procedure are based. This assumption is based on the requirement of the hazard ratios being constant over time or that the hazard for one individual is proportional to the hazard for any other individual. This proportionality constancy is independent of time. The test of proportionality showed clearly that the dependent covariate was statistically non-significant as the global test chi ^2^ was 1.23, df=4 and p=0.873, an indication of the constancy of hazard over time. This result indicated that the model did not violate the proportionality assumption. So, the appropriateness of the use of the model in the analysis was confirmed. Moreover, interaction in the model analysis showed that these interaction terms had no significant effects on the performance of the model.

## Discussion

A range of factors contributes to global and regional differences in cervical cancer incidence and mortality. Determining the drivers of these variations is complicated and there have been no comprehensive studies looking at this to date. However, stage, clinical features and quality of treatment are the most likely accepted explanations for these international differences. ^12^ Cervical cancer survival mainly depends on early detection and effective treatment modalities. Thus, by examining this survival through the eyes of prevention and control of the disease at diagnosis, one can assess and evaluate potential covariates with the most impact on patient survival. This study focused on cancer stage at diagnosis as the most important potential predictor covariate of survival. The result showed that these patients were relatively old, married, unemployed, illiterate, urban and belonged to non-Arab descent African groups. Cervical cancer, in Sudan, is described as advanced at presentation and grade, aggressive and invasive squamous cell carcinoma and moderately to poorly differentiated cells leading to poor survival. Several previous studies reached the conclusion of the disease as being invasive and advanced at presentation. ^12^^,^^19- 21^ The stage at diagnosis is much related to survival and cancer survival analysis measures this relationship and the effectiveness of the health care system.

This study showed clearly that advanced cancer stage presentation at diagnosis had a significantly negative impact on survival outcomes compared to the early stage. This conclusion is in agreement with previous studies in different developed and developing countries. ^14^^,^^16^^,^^19^^,^^21- 24^ Cancer survival is measured as a proportion of cancer patients who remained alive after a specific period, usually 5-years. However, this cancer survival measure is fundamentally influenced by stage, age, treatment therapy and if it is preventable and curable. Cervical cancer is preventable and relatively curable if detected at an early stage though most cancer cases are diagnosed at a late-stage in low- and medium-income countries and Sudan as shown in this study. ^6^^,^^25- 27^ Late-stage diagnosis is correlated with low survival rates, as well as complicated treatment, poor prognosis and survival outcome. ^28- 30^

This study demonstrated not only that late-stage cancer diagnosis influences survival negatively but, also, how each predictor covariate affects the slope of the survival curve using Cox regression analysis. In a four step elimination process of confounding factors, the results confirmed strongly that stage, chemotherapy and radiotherapy were the most likely predictor covariates of survival times. This result was in agreement with a recent study in Saudia Arabia. ^31^ Though the late cancer stage at diagnosis has proven to be closely related to poor survival, there are other factors associated with low survival rates such as socio-demographic, cultural, and economic characteristics of the patient, and histopathological features of the tumor. ^12^

Aside from the impacted survival rate, diagnosis of cervical cancer at an advanced stage has been explained by delays in diagnosis at presentation and initiation of treatment. ^12^ For cervical cancer, effective control measures are generally available and affordable. This disease can be, to a large extent, prevented by vaccination against HPV infection and by screening and treating pre-cancerous lesions. Other than this, early detection of cervical cancer is imperative to improve treatment outcomes. Assessment of the study conclusion should be interpreted with caution since the study was based on retrospectively routinely collected data from one referral hospital with the largest registration of cancer patients in the country. It does not include all data of cervical cancer patients in the country and is limited by the type of available data. Due to the huge differences in settings, it is prudent not to extrapolate from one experience in developed countries to others in developing countries.

## Conclusion and recommendations

The results of this study suggest that the cervical cancer stage at diagnosis and certain treatments are the most important factors impacting patient prognosis and survival outcome. The evidence presented has shown the complexity of determining what drives most variations in cancer outcomes between nations. It is most likely all steps the cancer patient takes when seeking medical care contribute to some degree to the differences in cervical cancer survival rates.

Cancer survival analysis can help in the diagnosis and treatment of cervical cancer and provide important information about where more effort should be directed. Early detection of cancer and vaccination of women against HPV infection provide tremendous opportunities for prevention, early diagnosis, more effective treatment and a higher probability of better survival and outcomes.

Government intervention to reduce the suffering of cervical cancer treatment is of vital importance by providing diagnostic and oncological services in all general public hospitals and introduction of oncology units in all state capital’s public hospitals. Early detection of cervical cancer should be the core of a proposed female cancer strategy through providing intensive and comprehensive vaccination, cervical cancer screening, and raising disease awareness among patients. This strategy needs to be closely linked to primary, secondary and tertiary care services.

## Data availability

### Underlying data

Zenodo: Elgoraish A. and Alnory A. cervical cancer dataset. http://doi.org/10.5281/zenodo.4399441 ^32^

This project contains the following underlying data:

- Cervical cancer dataset

### Extended data

Zenodo: Elgoraish A. Cervical cancer form. https://doi.org/10.5281/zenodo.4469654 ^33^

This project contains the following extended data:

- checklist.pdf
- consent form.pdf

Data are available under the terms of the Creative Commons Attribution 4.0 International license (CC-BY 4.0).
