# Supplementary material for: Associated predictor covariates of cervical cancer stage and impact on survival at Khartoum oncology hospital, Sudan
Source: F1000Res. 2022 Oct 7;10:114. Originally published 2021 Feb 15. [Version 2] doi: 10.12688/f1000research.43590.2 (PMC9585357; doi:10.12688/f1000research.43590.2)
Supplement: Peer review history

# Peer review: Reviewer response for version 2

**DOI:** 10.5256/f1000research.139379.r152671

**Published:** 2022-10-20

**Authors:** Elvynna Leong, Ong Sok King

**Affiliations:** 1 Faculty of Science, Universiti Brunei Darussalam, Jalan Tungku Link, Brunei; 2 NCD Prevention Unit, Ministry of Health, Commonwealth Drive, Brunei

No further comments to make.

Is the work clearly and accurately presented and does it cite the current literature?

Yes

If applicable, is the statistical analysis and its interpretation appropriate?

Partly

Are all the source data underlying the results available to ensure full reproducibility?

Yes

Is the study design appropriate and is the work technically sound?

Yes

Are the conclusions drawn adequately supported by the results?

Yes

Are sufficient details of methods and analysis provided to allow replication by others?

Partly

Reviewer Expertise:

Statistics, Public Health

We confirm that we have read this submission and believe that we have an appropriate level of expertise to confirm that it is of an acceptable scientific standard.

# Peer review: Reviewer response for version 1

**DOI:** 10.5256/f1000research.46657.r147507

**Published:** 2022-09-06

**Authors:** Elvynna Leong, Ong Sok King

**Affiliations:** 1 Faculty of Science, Universiti Brunei Darussalam, Jalan Tungku Link, Brunei; 2 NCD Prevention Unit, Ministry of Health, Commonwealth Drive, Brunei

This paper is very meaningful, especially for Sudan, as there are currently not many studies published from the Africa region or Sudan on cervical cancer survival. However, there are a few key components that require further clarification or analysis from the researchers.

**Title**

 The researchers aimed to examine predictors associated with cervical cancer stage at diagnosis and its impact on cancer patient prognosis and survival. However, the title of the paper does not state the word “stage”.

**Methods**

 Data collection and sources

- The authors stated a study period of 2011-2015 in the study design and follow-up in 2016. What is the earliest year of diagnosis? Were the participants recruited diagnosed during the 2011-2015 period only? Also, please give more details on the follow-up period. How long was the follow-up period? Is it up to December 2016?

Statistical analysis

- The statement “ *Statistical methods such as chi ^2^, Kaplan-Meier, log-rank test and Cox regression were used to find out most prognostic factors associated with cancer disease*” is not entirely correct. This needs to be revised. Explain why you used chi ^2^, Kaplan-Meier, log-rank test and Cox PH regression separately.
- " *Log-rank test for equality*." We suggest adding more detail on ‘equality’.

**Results**

 Presentation of results needs to be improved, such as

- Paragraph 1 in Descriptive statistics: removing **at **in " *(SE=0.88, at 95%CI=55.17-85.65)*".
- Paragraph 1 in Descriptive statistics: rewriting *“majority 82.9% ≥45 years old*”.
- Table 2: It is very important to keep the statistics consistent in tables, such as p-values of the log-rank test should be kept to 3 decimal places.
- Table 2: We suggest replacing 0.000 with <0.001, which is more commonly used in literature.
- Table 2: For Treatment, each p-value from the log-rank test is compared with no treatment?
- Table 2: On mean of survival time, to label months in the table.
- Regression analysis: " *(chi ^2^=33.49, df=3, p=0.000)*". Is chi ^2 ^=33.49 or 31.27, as indicated in Table 2?
- Regression analysis: " *The chi ^2^ was 7.91, df=1 with p=0.004 ( Table 2).*" **p-value is 0.005 (rounded up to 3 d.p).
- Regression analysis: “ *The graph of the two survival functions curves were statistically equivalent of the two groups*”. The p-value of 0.005 did not indicate statistical equivalence.

Table 1

- There were indications that geographical locations were significantly associated with stage of diagnosis, further discussion on this could be helpful.

For the survival analysis, was there a breakdown by 1-year or 3-years and 5-years survival rates? Was there any comparison or benchmarking of the findings or survival rates with other similar studies? It would also help to have some discussion on the potential explanations for these findings and to suggest for further studies. The researchers could identify the limitations of the study e.g. duration of treatment or any delay in treatment was not analyzed in the study.

 Some grammar problems should also be paid attention.

Is the work clearly and accurately presented and does it cite the current literature?

Yes

If applicable, is the statistical analysis and its interpretation appropriate?

Partly

Are all the source data underlying the results available to ensure full reproducibility?

Yes

Is the study design appropriate and is the work technically sound?

Yes

Are the conclusions drawn adequately supported by the results?

Yes

Are sufficient details of methods and analysis provided to allow replication by others?

Partly

Reviewer Expertise:

Statistics, Public Health

We confirm that we have read this submission and believe that we have an appropriate level of expertise to confirm that it is of an acceptable scientific standard, however we have significant reservations, as outlined above.

# Response

**Published:** 2022-10-04

**Authors:** Amanda Elgoraish

**Affiliations:** Goverment, Sudan

Some of your comments and suggestions have been taken care of in the text while others are clarified as below:

 The study period is called 5-years survival, 2011-2015, starting from 1 ^st^ Jan 2011 till 31 ^st^ Dec 2015. The follow-up was passively collected from participants' medical records and active follow-up during the year 2016 by contacting lost-to-follow-up participants to confirm their life status (dead or alive).

 The log-rank test for equality was intended to compare between different groups of covariates and to confirm their statistical significance differences.

 The survival tools of analysis were used to explain data analysis to suit descriptive and analytical sections and meet the requirement of each tool of analysis.

 Table 2: all p-values of the log rank test are kept to 3 decimal places.

 Table 2: for treatment each p-value of the log rank test basically is treatment and no treatment.

 Table 1: the location result (east and west) included p-values which were insignificant in the univariate model, but significant in multivariate model. It was also insignificant in the final regression model. The study was intended to examine cases in a referral hospital in Khartoum State. So it is difficult to make final conclusion on a very small dataset of participants outside Khartoum State which was beyond the scope of the study objectives.

 The study focused on 5-years survival (standard period for cancer survival analysis) and comparison with other short periods was not within the scope of the study.

 The study discussed other explanations for survival of cervical cancer patients in developing countries despite the paucity of similar studies. It refers to most recent published studies. Delay was mentioned as one of the most plausible explanations for cervical cancer patients' stage at diagnosis. We are in the final stage of presenting our new article on associated predictor covariates at diagnosis focusing on the delay as the most important factor.

 The study explained clearly its limitation based on availability and quality of data collected from participants' medical files and other sources.

# Peer review: Reviewer response for version 1

**DOI:** 10.5256/f1000research.46657.r84246

**Published:** 2021-05-24

**Authors:** Nazik Hammad

**Affiliations:** 1 Department of Oncology, Queen's University, Kingston, ON, Canada

Overall, the work is presented well. Some minor details that could be addressed:

 The introduction is too long and has number of repetitions. It needs to be shortened and made succinct.

 I recommend replacing developing and developed countries with the more current terms: low-and-middle-income countries ( **LMIC**) and high-income countries (HIC).

 It is best to situate this article in the context of the WHO cervical cancer elimination strategy launched 2020; the 90-70-90 targets for 2030 especially in the recommendation section where Sudan should strive to achieve the WHO cervical cancer elimination targets by 2030. These are measurable targets for the Ministry of Health in Sudan and various stakeholders.

 The last sentence in the discussion about the prudence of “not extrapolating from one experience in developed countries”, while correct, does not have relevance to the paragraph. I would suggest removing it.

 The study design is appropriate. Outcome research is desperately needed in LMIC to inform policy and measure that need to be taken to improve access to cancer care across the continuum.

 I have reviewed the methodology, the consent form and the follow-up and they have sufficient details. However, it would be of added benefit if the following could be addressed:

- Is there a different intake form that has the employment status and other sociodemographic variables?
- The authors may wish to explain what is the category “other tribes” that are neither non Arab descent Africans  or Arab descent Africans mean. This constitutes 11% of the cohort.
- It would add to the value of the research if a sentence can be added about how consent was obtained from women who cannot read and write?

The statistical analysis is very well done; however, I have the following questions:

- Has the chemotherapy and radiotherapy use been defined by stage? e.g., how many patients with advanced stage received chemotherapy versus those with early stage?
- The data shows that 57% of patients received chemotherapy. Was there any correlation between receiving chemotherapy and residing in Khartoum versus peripheral areas, between receiving chemotherapy and education or ethnic origin?

The data supports that access to early diagnosis and treatment is needed to improve survival. As such the conclusion is supported by the results. While this is not surprising, it is important for future planning of health services to document this in Sudan.

 The authors’ use of employment data as an indicator of socioeconomic status (SE) should be listed as a limitation as not all unemployed women in Sudan are of similar SE status especially in this cohort of older women. Household income and area of residence are likely to be indicative of socioeconomic status which may influence access to care. This data might have been difficult to collect but this should also be acknowledged.

 The data does not provide indications as to the reasons for lack of access to chemotherapy. Lack of treatment details such as completion of a full course or radiotherapy and chemotherapy is also a limitation.

 Health equity and cancer disparities should be highlighted. The data revealed that these patients are 62.2% non Arab descent African. Is this reflective of the general population in the area or is there more prevalence of cervical cancer among non Arab descent Africans? This is of relevance when planning prevention (vaccination), screening, treatment interventions by targeting the most vulnerable population.

 The conclusion could be strengthened by calling further research looking into barriers to access to treatment. For example, this current cohort could be further interrogated in the future by investigating details of initiation and completion of treatment including chemotherapy and radiotherapy

 Overall excellent effort in a much needed area of health services research. I believe the paper has academic merit, but I ask for a number of small changes to the article and response to some queries.

Is the work clearly and accurately presented and does it cite the current literature?

Yes

If applicable, is the statistical analysis and its interpretation appropriate?

Yes

Are all the source data underlying the results available to ensure full reproducibility?

Partly

Is the study design appropriate and is the work technically sound?

Yes

Are the conclusions drawn adequately supported by the results?

Yes

Are sufficient details of methods and analysis provided to allow replication by others?

Partly

Reviewer Expertise:

NA

I confirm that I have read this submission and believe that I have an appropriate level of expertise to confirm that it is of an acceptable scientific standard.

# Response

**Published:** 2021-06-30

**Authors:** Amanda Elgoraish

**Affiliations:** Goverment, Sudan

Your comments concerning length of introduction, using LIMC instead of developing and developed countries and the last sentence of limitation paragraph have been taken notice of and appreciated.

 The suggestion of outcome research is commended but it is beyond the purpose of this study. However, implementation research is more urgent to assess efficacy and effectiveness of intervention and early detection programmes.

 Consent and follow-up was conducted by phone to collect vital status data (dead or alive) after explaining purpose of the interview and having verbal consent. Missing socio-demographic data were also, obtained during the interview to complete already collected data from patients medical files and diagnosis profiles. Other tribes indicate to participants who were Sudanese but did not belong to any of the known Sudanese tribes. They are most likely belong to non-Sudanese foreign ethnic groups.

 Participant were classified as early or advanced stages at diagnosis and majority of them were at the advanced stage. Thus, most of participants who received chemo and radio therapies are most likely belong to this advanced stage. The study focus was on stage at diagnosis as the major determinant of survival. Other socio-demographic factors, residence state and rural/urban centres were considered insignificant confounding variables.

 Household income and area of residence are proxy of socio-economic status and may influence access to care but the final conclusion of data analysis after removal of confounding variables and interaction terms affirmed that only stage at diagnosis and chemo and radio therapies are the effectors of cervical patient survival and outcome.

 The majority of participants of cervical cancer disease were from the non-Arab decent African tribes is an indication which can be of great help in planning vaccination and screening programmes.

 Suggested future research on barriers to treatment is commended but barriers to early detection is more urgent and appropriate in the short term future perspective.

 Sufficient details of methods and analysis and source data are available and adequate for further reproducibility by looking into data availability section of the article. 

 Amanda Elgoraish

 Corresponding author
